# Genetic testing of common and rare variants in dementia patients from a memory clinic: Dementia-related genetic testing in memory clinic

**DOI:** 10.1186/s13195-025-01854-z

**Published:** 2025-10-14

**Authors:** Itziar de Rojas, Marc Hulsman, Niccoló Tesi, Rosalina M.L. van Spaendonk, Jetske van der Schaar, Janna I.R. Dijkstra, Wiesje M. van der Flier, Fred van Ruissen, Philip R. Jansen, Marcel T. Reinders, Mieke M. van Haelst, Yolande A.L. Pijnenburg, Maria Victoria Fernandez, Agustín Ruiz, Henne H. Holstege, Sven J. van der Lee

**Affiliations:** 1 Ace Alzheimer Center Barcelona, Universitat Internacional de Catalunya, Barcelona, Spain; 2 Network Center for Biomedical Research in Neurodegenerative Diseases, CIBERNED, National Institute of Health Carlos III, Madrid, Spain; 3 Luxembourg Centre for Systems Biomedicine (LCSB), University of Luxembourg, Esch-sur-Alzette, Luxembourg; 4 Genomics of Neurodegenerative Diseases and Aging, Human Genetics, Vrije Universiteit Amsterdam, Amsterdam UMC location VUmc, Amsterdam, The Netherlands; 5 Alzheimer Center Amsterdam, Neurology, Vrije Universiteit Amsterdam, Amsterdam UMC location VUmc, Amsterdam, The Netherlands; 6 Amsterdam Neuroscience, Neurodegeneration, Amsterdam, The Netherlands; 7 Delft Bioinformatics Lab, Delft University of Technology, Delft, The Netherlands; 8 Department of Human Genetics, Research & Diagnostic Center ADORE, Amsterdam UMC, Amsterdam, the Netherlands; 9 Department of Human Genetics, Amsterdam UMC, Amsterdam Reproduction and Development Research Institute, University of Amsterdam, Amsterdam, Netherlands; 10 Clinical Genetics, Department of Human Genetics, Amsterdam UMC, Amsterdam, The Netherlands; 11 Department of Complex Trait Genetics, Center for Neurogenomics and Cognitive Research, VU University, Amsterdam, The Netherlands; 12 Amsterdam Reproduction and Development, Amsterdam UMC, Amsterdam, The Netherlands; 13 Emma Center for Personalized Medicine, Amsterdam UMC, Amsterdam, The Netherlands; 14 Glenn Biggs Institute for Alzheimer’s and Neurodegenerative Diseases, University of Texas Health Science Center, San Antonio, TX USA

**Keywords:** Genetic testing, Alzheimer’s disease, APOE, Genetic risk score

## Abstract

**Background:**

Many types of dementia have high heritability, which creates opportunities for DNA diagnostics. Clinicians sporadically test for causal genetic variants. However, in addition to causal genetic mutations, an increasing number of both common and rare risk factors are being identified, especially for Alzheimer’s disease (AD). Here, we describe and evaluate diagnostic performance of combining genetic risk factors for AD to assist memory clinic clinicians.

**Methods:**

A retrospective analysis of 998 consecutive patients (mean age 62.1, 40.3% females, 63.3% dementia) was conducted over 2.5 years in a Dutch memory clinic. The patients underwent a complete genetic risk assessment, including whole-exome sequencing and array genotyping. We examined known pathogenic genetic variants for all dementia types and their correlation with clinical diagnoses. We evaluated a combined genetic score (GS) based on all genetic risk factors for AD - namely *APOE* genotypes, candidate risk rare variants in 11 genes, and a polygenic risk score (PRS) based on 82 common variants. Then, we analyzed the discriminatory characteristics of the GS.

**Results:**

Causal pathogenic variants were rare, present in 3.4% of individuals, but genetic testing would have altered the diagnosis in over half of the carriers. Candidate rare risk variants were more common, identified in 31.6% of patients. Both *APOE* genotypes and the PRS were independently associated with AD, and gene-specific interaction was found between *TREM2* and AD-PRS (β = -1.16, *p* = 0.015). Patients with a high GS were 7 times more likely receive an AD diagnosis compared to those with a low GS (*p* = 2.5E-07).

**Conclusion:**

Overall, this study highlights the potential of integrating genetic risk factors into clinical practice to enhance AD diagnosis, though the improvement in diagnostic accuracy was moderate. The findings underscore the importance of genetic testing in diagnosis while also recognizing its limitations.

**Supplementary Information:**

The online version contains supplementary material available at 10.1186/s13195-025-01854-z.

## Background

Dementia, a prevalent and costly global health concern, imposes a significant burden on affected individuals, caregivers and society. Accurately diagnosing the underlying causes of dementia is challenging but essential for effective care and treatment strategies [1].

While clinical diagnoses traditionally rely on neuropsychological assessments and imaging, the role of genetic factors in dementia is gaining recognition. Familial forms for Alzheimer’s disease [1, 2] (AD) and other heritable forms of dementia have been linked to specific causal pathogenic variants, such as in the *APP*, *PSEN1*, and *PSEN2* genes [3–6]. Identification of genetic factors has implications for family members and may influence clinical decision making [7]. This genetic information, especially in the presence of high-penetrance pathogenic variants causing monogenic diseases, is crucial for explaining disease origins in patients and predicting development in healthy relatives [8]. Through technological advancements genetic testing will become cheaper, enabling more patients to be screened [9]. However, strict criteria limit genetic testing to specific patients with a strong family history or early-onset dementia, leaving many carriers of such mutations untested.

The majority of sporadic AD cases have a large genetic component. Both common and rare genetic risk factors are identified and explain part of the heritable risk of AD [10]. Genome-wide association studies (GWAS) are instrumental in identifying susceptibility loci for complex diseases. Currently, over 80 common genetic risk factors associated with AD-risk in populations of European ancestry [11–13] have been identified using GWAS. Moreover, GWAS datasets can be used to determine a polygenic contribution of common single nucleotide polymorphisms (SNPs) that show disease association [12, 14] and disease progression [15, 16]. Polygenic risk scores [17] (PRSs) are designed to aggregate genome data into a single variable, indicating the genetic predisposition for a disorder or trait. These scores have been suggested as valuable tools for the selection of individuals for clinical trials targeting different traits (e.g. diabetes, cardiovascular disease, breast cancer) [18–20]. However, PRS do not include the effects of rare variants [21–23] preferably detected through by sequencing [24] techniques. Rare variants are often much more important in terms of disease risk. One of the best studied examples is the R47H mutation in *TREM2* [21, 22, 25] gene, with a similar [26] increased risk of AD as one copy of *APOE* ε4 allele —the most established genetic factor for AD [27, 28].

In sum, knowing the complete contribution of common and rare genetic factors to disease etiology may be valuable for some patients and their family. In a case of familial pathogenic variant, information and family planning options can already be provided for next generations. In the future genotype risk data can provide a patient-specific, time-independent risk profile that could be used to prioritize different diagnoses and maybe treatments. Therefore, we tested the potential of DNA-diagnostics in patients attending a specialized memory clinic. We tested both the monogenic causes of dementia and a genetic score (GS) that combines rare high-risk variants and common genetic risk factors for AD [29]. This screening for monogenic causes of dementia may show clinicians the beneficial effect of genetic testing in clinical care and with the GS we investigated if (in a care setting) the polygenic architecture of AD can discriminate patients with AD from patients without dementia or other types of dementia [11, 13, 24, 30, 31].

## Methods

### Cohort

In this retrospective study, we included all patients who visited the Alzheimer Center Amsterdam between the first of January 2010 and the first of July 2012 (2.5 consecutive years) and consented to research by participating in the Amsterdam Dementia cohort (ADC) (Medical Research Ethics Committees, Amsterdam UMC, location VUMC 2016.061, 2017.315). This study cohort was previously used to describe new clinical criteria for monogenic forms of dementia [32]. All patients underwent the same diagnostic trajectory [33]. All individuals received a standardized diagnostic workup which did not significantly change between 2010 and 2012 [32–34]. Briefly, this includes a medical and neurological investigation, neuropsychological assessment, brain magnetic resonance imaging (MRI) and if necessary cerebrospinal fluid (CSF) analysis. Diagnoses were made by consensus in weekly multidisciplinary meetings and based on established international clinical and research criteria for major dementias (e.g., NINCDS-ADRDA [35, 36] for AD, Lund-Manchester [37] for frontotemporal dementia (FTD), and McKeith criteria [38, 39] for dementia with Lewy bodies (LBD)). For mild cognitive impairment (MCI), Petersen’s criteria [40, 41] were used until 2012, after which the National Institute on Aging–Alzheimer’s Association (NIA-AA) criteria [42, 43] were adopted. As part of informed consent, patients were asked for permission to extract DNA from blood and to participate in genetic studies.

Four categories were delineated according to their clinical phenotype: (1) individuals characterized as either cognitively healthy or experiencing subjective cognitive decline (SCD), (2) MCI, (3) patients with AD alongside individuals with MCI who exhibited amyloid-positive in CSF, and (4) individuals diagnosed with other forms of dementia (such as FTD, LBD or vascular dementia (VD)), as well as those with primary psychiatric disorders (PPD). It is important to note that PPD, despite presenting cognitive symptoms, are not considered a form of dementia, so we treated them separate in our study.

### Genetic analyses

We included all patients for which array genotyping (with Trans-Omics for Precision Medicine (TOPMed) imputation [44–46] whole-exome sequencing (WES) and functional annotation, *C9orf72* repeat lengths, and *APP* duplication status were available (see Supplementary information for more details).

### Identification of (likely) pathogenic genetic variants

We extracted genetic variants in the exons and flanking regions (+/- 6 basepairs) from 54 genes related to monogenic dementia for each patient [46] (Supplementary Table 1). All variants were annotated for pathogenicity using the Alissa Interpret^®^ software (Agilent Technologies - v5.4.0). Intronic variants were assessed using webtool SpliceAI (https://spliceailookup.broadinstitute.org/). Only variants with population frequencies < 1% were considered. Variants that were unclassified after initial filtering steps based on frequency, were classified independently by a trained researcher and a clinical molecular geneticist. Variant classification followed the guidelines published by the American College of Medical Genetics (ACMG) and Genomics and the Association for Molecular Pathology in 2015^47^. The classification is based on the level of evidence available for each variant (I-V). Variants that were classified by one or both as class III/IV/V were discussed and a consensus was reached. We supplemented our test panel with tests done in clinical care based on the clinical presentation of the patient. Because of slowly progressive muscle weakness and spasticity one patient was tested for a gene panel of movement disorders (pathogenic variant found in *SPAST* gene) and one patient was tested for HTT repeat expansion because of subtle chorea (*HTT*-repeat expansion found of 44 repeats).

### Rare risk variants for alzheimer’s disease

We extracted candidate risk rare variants (population minor allele frequency (MAF) < 1%) in AD associated genes based on a previous publication [24] including risk genes (*ABCA1*,* ABCA7*,* ATP8B4*,* TREM2 and SORL1)* and candidate risk genes (*ADAM10*,* SRC*,* RIN3*,* CLU*,* ZCWPW1* and *ACE*) using the WES data filtering for different deleteriousness thresholds by LOFTEE [48] score using Variant Effect Predictor (VEP) [49] annotator (loss-of-function (LOF) variants) and Rare Exome Variant Ensemble Learner (REVEL) [50] score for missense variants (see Supplementary methods & Supplementary Table 2). *SORL1* LOF variants were classified as causal (monogenic) for consistency as in previous studies [32, 51]. In contrast, *SORL1* missense variants were categorized as rare risk variants.

### Common variants, PRS calculation

We calculated a weighted individual AD-PRS using the genome-wide (GW) significant SNPs (MAF > 1%) and effect sizes previously published by Bellenguez et al. [11] (Supplementary Table 3). We decided not to include *APOE* within the AD-PRS itself, as the major common genetic risk factor for AD [28, 52] to allow us to analyze its behavior separately among carriers and non-carriers. The PRS was generated following the standard approach multiplying the genotype dosage of each risk allele for each variant by its respective weight expressed as beta coefficient and then summing across all variants [53–55]. The PRS is expected to be higher in cases than in controls, indicating a higher genetic risk for the disorder, but the difference in mean PRS between case and control samples may be small. The PRS calculation was implemented with R-statistical software (R 4.4.2).

### Interaction between AD-PRS and rare variants

To assess whether the effect of rare variants interacts with the common AD-PRS, we performed logistic regression models including an interaction term. We further explored gene-level effect using models of the form: Status ~ AD-PRS*Carrier-gene where ‘Carrier-gene’ is a binary indicator for each rare variant gene.

### Statistical analysis

We included four non-exclusive different categories of individuals based on the genetic profile for different analyzes; (1) Monogenic carriers defined as a patient with a likely pathogenic or pathogenic variant (or two, in case of autosomal recessive inheritance) in a gene related to neurodegenerative diseases. Monogenic carrier’s vs. the others subjects were compared in terms of age at entry, sex, family history of dementia and *APOE* genotype (using the two-proportions z-test in R). In addition, we also compared the clinical diagnosis with the genetic diagnosis as a reference for these subjects. After that, we excluded this group of individuals for the analysis. (2) Carriers of candidate rare risk variants in moderately penetrant AD susceptibility genes (Supplementary Table 2). (3) AD-PRS (without *APOE* and MAF > 1%). We divided the AD-PRS into quintiles based on the SCD (healthy individuals) population. The quintiles were defined as very low, low, reference, risk and high-risk respectively. Subjects with extreme values on the PRS and age (low/high PRS and early/late age at entry (early ≤ 65, late > 65 years)) were compared in terms of age at entry, PRS, sex, family history of dementia and *APOEε4* carriers (using the two-proportions z-test in R). Then, we performed a multinomial logistic regression model with the AD-PRS (scaled and standardized) as predictor, adjusted by age, to predict the probabilities of the different possible clinical diagnosis (SCD, MCI, AD and other dementias) taking SCD as a reference. Logistic regressions between SCD and AD with the PRS adjusted by age, sex and *APOEε4* were performed. We also included a (4) analysis for *APOEε4* carriers and non-carriers. We use Venn diagrams to show the overlap between the PRS (high risk/very low) groups, being a rare variants carrier and an *APOEε4* carrier. Finally, Chi-square test was used to evaluate the presence of significant differences between the phenotype groups in the categories 2, 3 and 4 (rare, PRS and *APOEε4* respectively).

### Genetic profile score

We generated an individual ‘*genetic score (GS)’* by combining three genetic components: the common variant AD-PRS, the *APOE* effect and the rare variant PRS. Each component was calculated using the standard PRS approach, multiplying the genotype dosage of each risk allele by its respective weight (beta coefficient) and summing across all variants. For the rare variant block, effect sizes were obtained from Holstege et al. [24] (see variants included in Supplementary Table 2), accounting for individual age to distinguish between early/late-onset effect. For the two *APOE* markers, we used the effect sizes reported in de Rojas et al. [12]. Then, the GS was computed as the standardized sum of these three components. A stratification was made into quintiles (q1=-1, q2=-0.5, q3 = 0, q4 = 0.5, q5 = 1) based on the distribution observed in SCD individuals. Odds ratios (OR), sensibility, specificity and positive/negative predicted values (SE, SP, PPV & NPV) were calculated using the GS as predicted values and the clinical diagnoses (SCD and AD) as a gold standard. For negative GS values, SCD was considered as the correct outcome. non-SCD or AD phenotypes were not included for these analyzes. Next, we tested the association with age at entry in AD cases with the GS and AD-PRS adjusted for sex and *APOEε4* through linear regressions.

### Discrimination analysis

Finally, we calculated the area under the receiver operating characteristic curve (AUC) by logistic regression models including the continuous GS, age, sex and the interaction between the common AD-PRS and carrying a rare *TREM2* variant for discriminating the observed SCD/AD and dementia AD/nonAD status using the fitted() function and ‘ROCR’ package in R-statistical software. To compare the AUC performance of the models, we used the DeLong Test with the roc.test() function and ‘pROC’ package in R. Then, SE, SP, PPV and NPV were calculated using the confusion matrix based on the predicted and actual values from each logistic regression model. All analyses were done in R (version 4.4.2).

## Results

In total 1,138 patients visited the clinic through this period and gave informed consent for research. 45 patients (4%) who did not consent to DNA research were not included. No DNA was available for 60 patients (5.3%) and 11 (9.7%) were excluded because of poor DNA/sequencing quality. Details regarding the subjects’ demographics and clinical characteristics are summarized in Table 1. The AD phenotype comprised 36.3% of the individuals (48.1% females) with higher age at entry (65.6 years), greater prevalence of family history of dementia (44.8%), and higher proportion of *APOE*ε*4* carriers (64.6%).

A summary of all analysis is shown in Table 2 and detailed results are in the following paragraphs.

**Table 1:** Demographics characteristics of the participants in this study. SCD: subjective cognitive decline, MCI: mild cognitive impairment, AD^a^: alzheimer’s disease and MCI with positive beta amyloid in cerebrospinal fluid (CSF), other: other dementia and primary psychiatric disorders. P-value: pearson’s Chi-squared test

Cohort demographics (*n*,%)	SCD (*n* = 219)	MCI (*n* = 87)	AD^a^ (*n* = 362)	Other (*n* = 328)	Total (*n* = 998)	*P*-value
Age at entry (years, SD)	57.8 (9.3)	64.3 (7.5)	65.6 (7.2)	60.6 (9.0)	62.1 (8.9)	2.5E-07
Gender (Females)	91 (41.6)	21 (24.1)	174 (48.1)	115 (35.1)	402 (40.3)	5.6E-05
Family history of dementia (% yes)	84 (38.4)	26 (29.9)	162 (44.8)	90 (27.4)	363 (36.4)	1.1E-05
Monogenic carriers	3 (1.4)	4 (4.6)	8 (2.2)	17 (5.2)	34 (3.4)	2.8E-04
Carrier of rare AD variants (Early/Late, ± 65 years)	67 52/15 (30.6)	28 15/13 (32.2)	130 55/75 (35.9)	90 53/37 (27.3)	315 175/140(31.6)	0.107
APOEε4 carrier	81 (37.0)	37 (42.5)	234 (64.6)	134 (40.6)	486 (48.7)	1.0E-12
CSF (Amyloid positive %)	166 (12.8)	60 (0)	308 (79.3)	253 (20.6)	787 (38.5)	< 2.2E-16

**Table 2:** This report represents results based on application of genetics in 998 individuals recruited in 2.5 years in a Dutch memory clinic, the Amsterdam VUMC center

Components of genetic profiles
**(1) Monogenic dementia; Patients with a monogenic cause of dementia** Genes found: AD - APP, PSEN1 and SORL1 FTD - MAPT, C9orf72, TARDBP, CTSF and GRN Other dementia - Huntington, CHMP2B, NOTCH3, NPC1, PRNP and SGP4 Results: 34 out of 998 (3.4%) individuals were carriers of a pathogenic variant in one of these genes.
**(2) Carriers of rare variants in moderately penetrant AD susceptibility genes** Genes analyzed (*n* = 11): *SORL1*,* TREM2*,* ABCA7*,* ATP8B4*,* ABCA1*,* ADAM10*,* SRC*,* RIN3*,* CLU*,* ZCWPW1 and ACE* Results: 306 out of 964 (31.7%) individuals were carriers one of these candidate risk variants and 58 (6%) carried 2 or more.
**(3) AD polygenic risk score** 82 variants from 77 loci combined (without *APOE*) Results: PRS was significantly associated with AD risk
**(4)** ***APOE*** **stratification** Results: 473 out of 964 (49.1%) individuals were *APOEε4* carriers. 47.5% of the ADs were *APOEε4* heterozygous and 17.8% were *APOEε4ε4* homozygous carriers.

### Pathogenic/Causal genetic variants

**Fig. 1:**
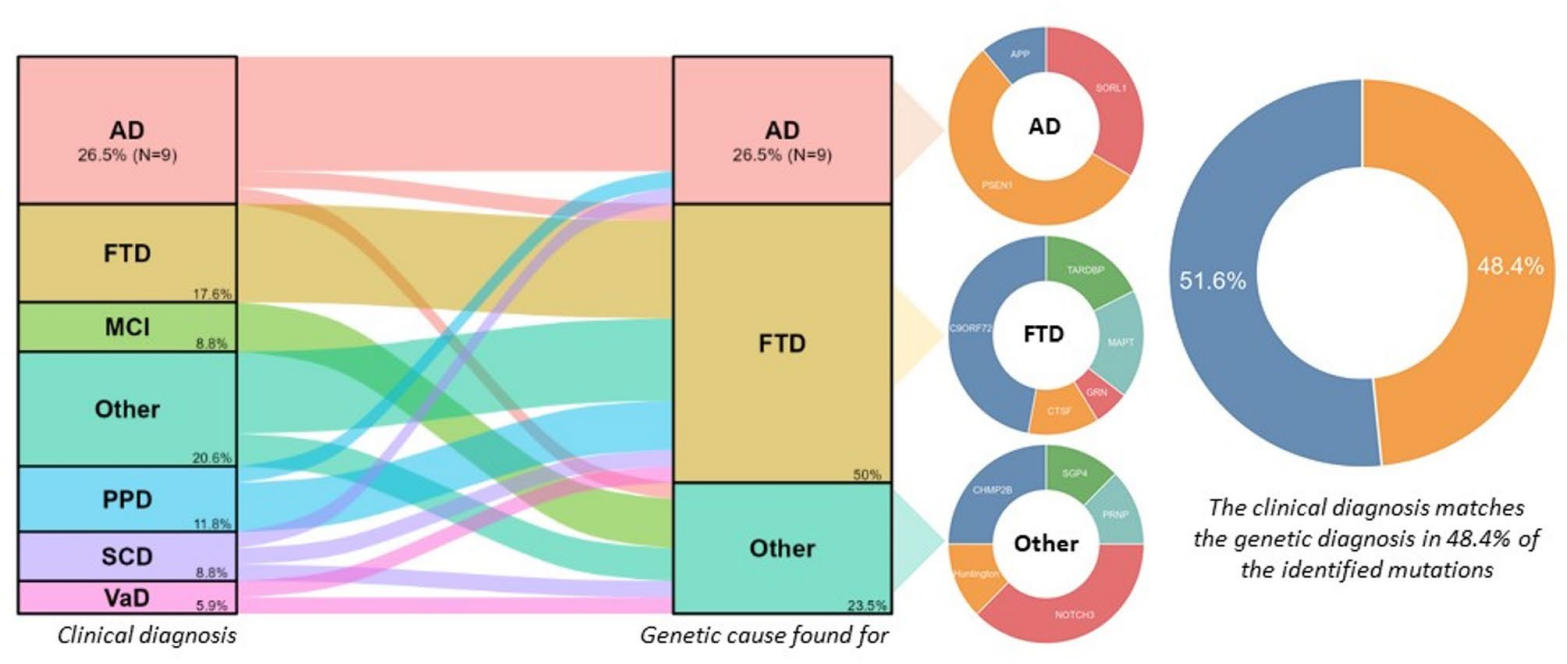
Transition plot and phenotype distribution of the pathogenic variants. The left figure shows the distribution and changes between the clinical diagnosis before genetic testing and the final diagnosis based on the genetics for the monogenic carriers. Pie charts present the proportion for the different pathogenic variants found in each phenotype as well as the percentage of clinical misdiagnoses if genetic testing had not been done (SCD were excluded for the misclassification calculation). AD: Alzheimer’s Disease, FTD: Frontotemporal Dementia, MCI: Mild cognitive Impairment, PPD: Primary Psychiatric Disease, SCD: Subjective Cognitive Decline, VaD: Vascular Dementia, Other: Other dementia

A total of 34 unrelated subjects were identified as having a causal genetic variant (category 1, monogenic dementia), corresponding to a prevalence of 1 in 30 patients (3.4%). Among these monogenic carriers, 9 individuals (26.5%) had a pathogenic variant in a gene associated with AD, 17 individuals (50%) had a pathogenic variant in a gene associated with FTD, and 8 individuals (23.5%) had pathogenic variants in genes associated with other types of dementia (Fig. 1, Supplementary Fig. 1).

Comparison between the clinical diagnosis and the genetic diagnosis based on pathogenic variants revealed that for 16 of 31 individuals (51.6%) the initial clinical diagnoses did not align with the genetic diagnoses (excluding the SCD cases (*n* = 3) from the calculation, Supplementary Table 4). As anticipated, carriers of pathogenic variants were significantly younger at the time of entry (*p* = 0.011). Additionally, these carriers had a higher, though not statistically significant, family history of dementia, a greater proportion of females, and more *APOEε4* non-carriers compared to the rest of the subjects (Supplementary Fig. 2).

**Fig. 2:**
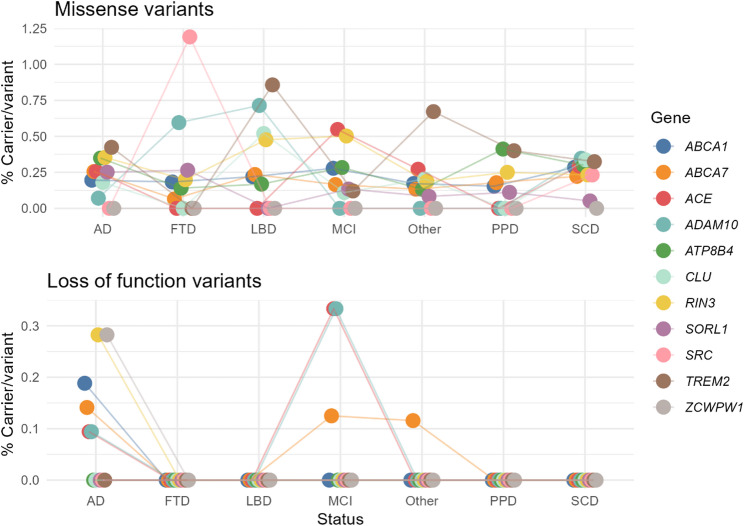
Distribution of rare AD-variants between the clinical phenotypes by gene. Carrier per variant percentage were calculated based on the number of carriers divided by the number of variants and the individuals in the phenotype group

We then examined the candidate rare variants in the 11 selected genes associated with moderate AD risk. We found that one out of three individuals carried a candidate rare variant: 306 subjects (31.7%) carried at least one, while 58 subjects (6%) carried two or more moderate-risk variants (Supplementary Fig. 3). A wide range of different variants was observed, with a total of 184 unique variants. Of these variants, 88% were missense variants and 12% were LOF or predicted to affect splicing (VEP) (Table 3, Supplementary Table 2) showing a dispersion in the proportion of carriers between the different clinical phenotypes (Fig. 2). Candidate rare variants were found in almost all genes within AD individuals. In contrast, the FTD and LBD phenotypes showed the highest carrier frequencies for missense variants in some genes such as *SRC*, *ADAM10*,* CLU* or *TREM2*, while no carriers were observed for LOF variants. Similarly, no LOF variants carriers were identified among PPD and SCD phenotypes. The most frequently observed single missense variants were chr19:1059056:G:A in *ABCA7* (nº carriers = 22), chr14:93142861:T: C in *RIN3* (nº carriers = 19), chr17:61601584:A: G in *ACE* (nº carriers = 15) and chr6:41029294:C: A in *TREM2* (nº carriers = 12). *TREM2* R62H was not included here due to the allele frequency filter (MAF < 1%) and *TREM2* R47H mutation (chr6:41129252:C: T) was observed in ten individuals. No individuals carrying both the R47H and R62H mutations in *TREM2* were identified.

**Table 3:** Selection and carriers of rare AD-variants based on the criteria defined in the methods section (category 2). The sample size was *n* = 964 individuals. The total is the sum of the carriers, considering that an individual can carry more than one variant

Genes	nº Variants	nº Missense	nº LOF	nº LOF Carriers	nº Missense Carriers
*ABCA1*	16	13	3	4	27
*ABCA7*	81	73	8	10	146
*ACE*	14	11	3	4	26
*ADAM10*	7	4	3	3	6
*ATP8B4*	17	17	0	0	48
*CLU*	11	11	0	0	21
*RIN3*	13	12	1	1	36
*SORL1*	9	9	0	0	13
*SRC*	2	2	0	0	2
*TREM2*	10	10	0	0	39
*ZCWPW1*	4	0	4	5	0
Total	184	162 (88.04%)	22 (11.96%)	27	364

**Fig. 3:**
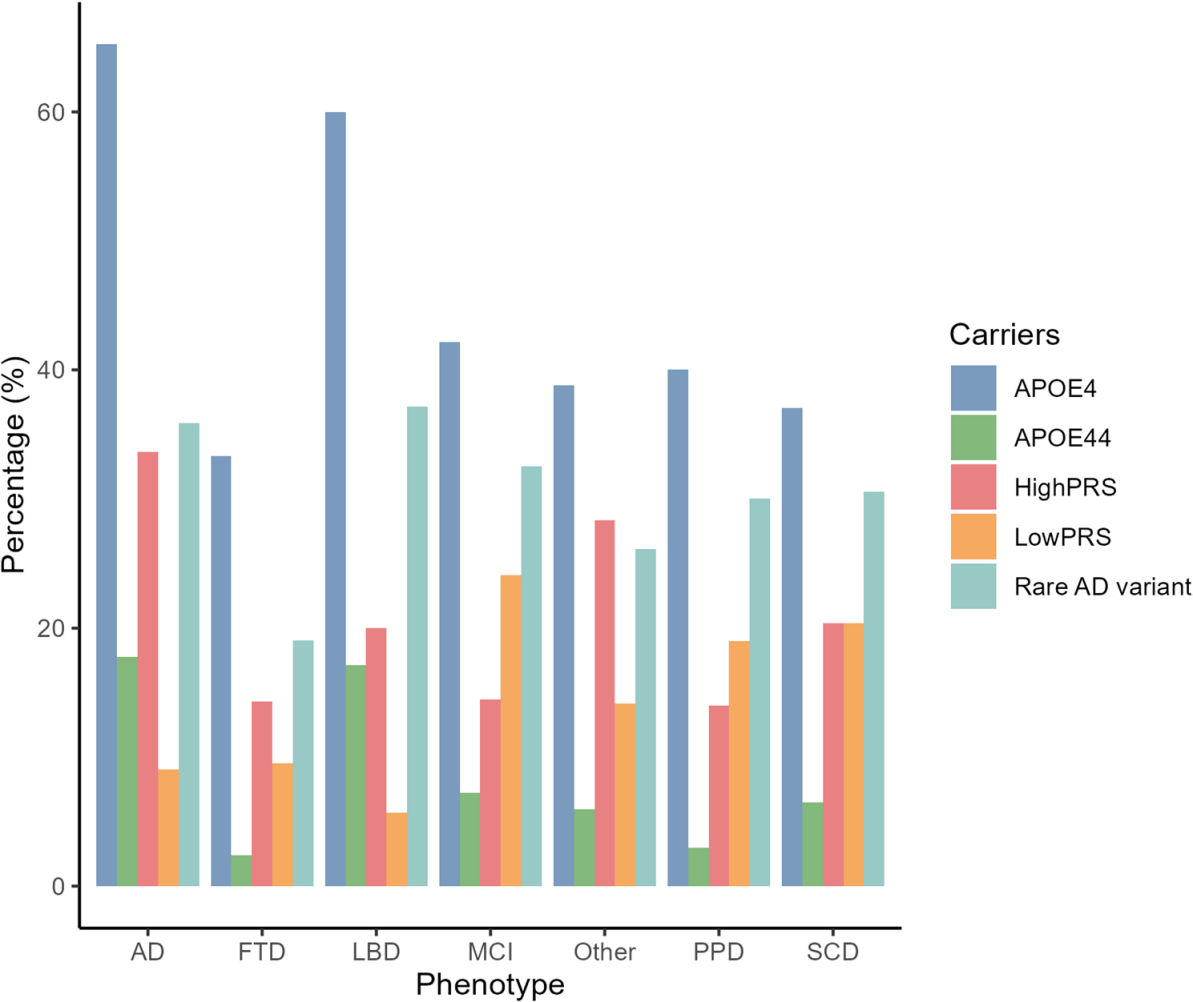
Percentage of individuals carrying *APOEε4*, high/low AD-PRS and candidate rare AD variants in each clinical diagnosis group. APOE4 = carriers of at least one *APOE*ε4 allele, APOE44 = *APOE*ε4 homozygous, AD = Alzheimer’s disease; LBD = Lewy Body dementia; FTD = Frontotemporal dementia; MCI = Mild cognitive impairment; Other = Other dementia; PPD = Primary Psychiatric Disease; SCD = Subjective cognitive decline; High/LowPRS = highest and lowest quintiles for common AD-PRS based on the SCD population

In our analysis of carriers of candidate rare variants associated with AD across different clinical phenotypes (Fig. 3), we observed that AD and LBD exhibited the highest prevalence, ranging from 35.9 to 37.1%. In contrast, FTD and other forms of dementia showed lower frequencies, ranging from 19.7 to 26.1%. Patients with a primary psychiatric diagnosis, MCI and SCD displayed intermediate frequencies with approximately 30-32.5% of individuals carrying rare variants. Notably, 26.5% of individuals carrying a causal variant also carried at least one rare variant. A comparison of demographic characteristics between rare variant carriers and non-carriers revealed no significant differences in age at entry, gender, family history, or *APOEε4* carrier status (Supplementary Fig. 4). The Chi-square test revealed a statistically significant difference in prevalence between the phenotype groups in *APOE ε4* (*p* = 2.1E-06) and a high PRS (*p* = 1.6E-03) indicating a different AD-risk distribution between the phenotypes (Fig. 3).

The interaction term between AD-PRS and rare-PRS was marginally significant (*p* = 0.039, p-adjusted = 0.06), despite the rare variant PRS alone not showing a significant main effect (Supplementary Table 5). Gene-specific interaction analyses revealed that the interaction was mainly driven by *TREM2* carriers, with a significant interaction effect (*p* = 0.015) and a negative coefficient (β = -1.16), indicating that the effect of the common PRS on AD risk is attenuated among *TREM2* rare variant carriers (Supplementary Fig. 5). This highlights the importance of considering gene-specific interactions when combining common and rare genetic risk components, as rare, high-impact variants may overshadow or alter the risk conveyed by common variants.

### Genetic score

A higher GS was significantly associated with increased odds of AD after correcting for age, sex, and the interaction between common AD-PRS and carrying a rare *TREM2* variant (OR = 1.82[1.48–2.23] per one unit increase in the GS, *p* = 7.78E-09). Notably, when restricting rare variant analysis to only five risk genes (*SORL1*,* TREM2*,* ABCA7*,* ABCA1 and ATP8B4*) the association slightly improved (OR = 1.97[1.60–2.44], *p* = 3.15E-10, Supplementary Table 6). Moreover, from GS quintiles 4 to 5 we saw a 1.9–2.7 fold significant increase in the AD risk respect to the reference group (Table 4, Supplementary Table 7). As expected, sensitivity was low in all scores, while specificity was ~ 80% in all quintiles. When we compared the extremes of the GS distribution (q1 vs. q5), individuals in the highest genetic risk quintile showed a 7-fold increase in odds of disease compared to those in the lowest quintile (*p* = 2.5E-07). The predicted performance of the GS was most pronounced at this comparison, reaching a sensitivity of 90% and specificity of 49%.

**Table 4:** Genetic score (GS) associations adjusted by age, sex and the interaction between common AD-PRS and carrying a rare *TREM2* variant taking score = 0 as a reference in SCD (*n* = 216) and AD individuals (*n* = 354). OR = Odds ratio, se = sensibility, sp = specificity, ppv = positive predicted value, npv = negative predicted value. Finally, we calculated the AUC to find the best discrimination model for clinical diagnosis of AD in SCD/AD and dementia AD/non-AD groups, and we observed a similar trend in both groups (Table 5). Although genetics alone showed moderate discriminative ability compared with age and sex, it is noteworthy that the combination of genetic and demographic factors led to a statistically significant improvement over age and sex alone (*p* < 5.91E-04, DeLong test). This suggests that genetic information provides additional predictive value (Supplementary Fig. 6D)

Score	Effect	SE	*P*-value	OR [95%CI]	SE %	SP %	PPV %	NPV %
q1 (-1)	-1.038	0.402	9.8E-03	0.35[0.16–0.78]	19.91	96.05	75.44	66.28
q2 (-0.5)	-0.354	0.329	2.8E-01	0.70[0.37–1.34]	20.37	87.85	50.57	64.39
q3 (0)	Reference category							
q4 (0.5)	0.652	0.304	3.2E-02	1.92[1.06–3.48]	29.66	80.56	71.43	41.13
q5 (1)	0.995	0.298	8.3E-04	2.70[1.51–4.85]	38.98	79.63	75.82	44.33
q1 vs. q5	1.979	0.383	2.5E-07	7.24[3.41–15.34]	90.79	49.43	75.82	75.44

**Table 5:** Area under the receiver operating characteristic curve (AUC) results. The Delong test was calculated by comparing it with the reference model (age + sex). GS = Genetic score, PRS: TREM2 = interaction between common AD-PRS and carrying a rare *TREM2* variant, scd = subjective cognitive decline, ad = alzheimer’s disease, se = sensibility, sp = specificity, ppv = positive predicted value, npv = negative predicted value

Model	Variables	AUC	*P*-value	DeLong’s *p*-value	SE %	SP %	PPV %	NPV %
SCD vs. AD	Age + sex	0.740	7.11E-26	---	86.4	47.7	73.0	68.2
	GS + PRS: TREM2	0.687	8.64E-12	0.083	85.6	34.7	68.2	59.5
	GS + PRS: TREM2 + Age + Sex	0.785	2.12E-10	5.91E-04	83.1	53.7	74.6	65.9
AD vs. non-AD	Age + sex	0.676	1.62E-14	---	71.2	54.7	64.1	62.5
	GS + PRS: TREM2	0.679	2.13E-15	0.912	68.4	57.9	64.9	61.6
	GS + PRS: TREM2 + Age + Sex	0.745	1.60E-14	6.82E-06	72.0	62.7	68.7	66.3

## Discussion

Our study describes how monogenic causal variants behave in a clinical population and evaluated the potential testing genetic risk variants for AD in a clinical memory setting. Previous studies have explored the use of genetic markers to diagnose dementia in clinical settings [56, 57] but not in a consecutive series of patients that all underwent testing irrespective of their diagnosis and all undergoing the same diagnostic trajectory. We found that identifying monogenic causes of dementia is important when diagnosing patients with dementia, as over half of the clinical diagnoses did not match the genetic diagnoses. For the genetic risk variants, we found a strong statistical association of AD diagnosis with *APOE* and common variant PRS. When combining all these factors and the rare PRS into a single genetic score, the improvement in diagnostic discrimination, although statistically significant, was only marginal. This limited gain likely reflect the fact that both *APOE* and rare risk variants are enriched in AD patients but also present across other diagnostic groups, thus reducing their utility in discriminate AD from non-AD cases.

Strengths of our study include that we studied a series of consecutive patients that presented at a memory clinic over the course of 2.5 years. Approximately 90% of all patients entering the clinic were genetically tested, and all patients underwent the same diagnostic trajectory. This minimizes participation bias and the effect that differential application of diagnostic tests may have had on the diagnosis of patients. Due to genetic analysis in large case-control studies of well-defined case series we gain increasing knowledge of genetic risk factors of dementia. However, integration of genetic tests in a diverse real-world setting is rarely pursued. Therefore, our results reflect the true added diagnostic benefit of genetic testing in clinical care. It is essential to mention that the Amsterdam Alzheimer Center specializes in young onset dementia, which has led to an overrepresentation of young patients [32]. As a consequence, there is referral bias and a higher proportion of individuals carrying pathogenic and other rare mutations compared to a typical memory clinic. To reflect the real-world setting, we also did not exclude family members or participants of non-European decent. This might have reduced the efficacy of the PRS (as this originates from European ancestry analyses), but increased the studies external validity. A limitation to the use of genetic risk scores, is that all discoveries have concentrated in AD. The number of loci associated with vascular dementia, LBD [30, 58, 59] and FTD [60, 61] are small and we therefore did not use these in this study.

Genetic cause of disease aligned with the clinical diagnosis in only 48.4% of the pathogenic carriers, making obvious the importance of genetics in these individuals. This clearly highlights the importance of genetic testing for causal factors of dementia and the limitations of clinical diagnoses of underlying pathology. Genetic testing can be implemented in clinical care by using clinical criteria for genetic testing derived from the same dataset [32]. These criteria ensure the patients at highest risk are tested. The misdiagnosis of pathology based on clinical symptoms are also likely present in ‘sporadic’ forms of dementia. It was already shown in pathology studies that some risk factors discovered in AD-GWAS may in fact exemplify risk factors for limbic-predominant age-related TDP-43 encephalopathy (LATE) [62, 63] and clinical FTD may be a-typical frontal dementia. On top of this there may be co-pathology present, such as in LBD where half of patients also has AD in pathological series [64].

As the number of known common and rare genetic risk factors for AD continues to grow, there is increasing need to consider how these findings can be integrated into clinical care. In this study, we provide a comprehensive description not only of causal variants but also of rare variants and distribution of common risk factors in memory clinic population. Our findings align with recent prospective clinical work [65] which reported that 12.2% of EOAD patients carried rare variants in established AD risk genes. While they advocate for variant-level clinical interpretation based on OR thresholds and pathogenicity classification, our analysis reveals a significant interaction between the common AD-PRS and rare variant burden, specifically driven by *TREM2* rare variant carriers. This suggests that the presence of high-impact rare variants may attenuate or modify the contribution of PRS to AD. These findings emphasize that genetic risk is not merely additive; rather, interactions between rare and common variants can shape individual risk profiles in non-linear ways. Clinically, this highlights the need for integrated risk models that account for both rare and common genetic contributions, as individuals carrying highly penetrant rare variants (such as those in *TREM2*) may derive limited additional risk stratification from PRS alone. Future studies should explore whether similar modifying effects exist for other rare risk genes and how such interactions could inform precision medicine approaches. Together, these studies highlight the multifactorial and potentially oligogenic architecture of AD and support complementary strategies for integrating rare and common variants in both research and clinical contexts.

We proposed a GS that combines all genetic risk factors for AD (excluding the monogenic causes) in a single score. This greatly simplifies the patient’s risks but may smooth over fine-grained risk increases. We only included genome-wide common variants and rare variants identified in known risk genes in our GS [11, 24]; however, it is important to note that many of the rare variants should currently be considered candidate risk variants, as the available evidence does not yet support their clinical use or reporting back to patients. For example, for *SORL1* there is an active scientific debate on which variants to interpret as monogenic or candidate risk variant. We currently made the decision to include pathogenic variants in the monogenic group, while some missense variants may have similar effects [66–69]. The interpretation of *SORL1* variants is thus subject to the individual interpretations of researchers and clinicians and may change with accumulating evidence. While this approach improves replicability across populations [70] and reduces overfitting or inflated discrimination [12, 71], it inevitably limits the inclusion of potentially informative variants [58]. We chose not to include more variants as we find the discrimination in the non-European ancestry groups as important as the marginal improvement in AUC in European ancestry groups. By keeping the inclusion criteria clear, the GS can also easily be updated as novel rare variants are discovered and may be implemented population specific if GWAS data become available for populations with non-European ancestries. Our current approach aims to capture overall genetic burden but of course individual variant interpretation would be necessary in clinical settings. As mentioned, the improvement in patient discrimination was significant, but only small in absolute improvement of the AUC. We do observe that the discriminative value of the GS is higher when we consider the extremes [q1 and q5 groups; 42% of the patients]. The marginal increase in AUC and improved discrimination between AD cases and controls when considering the extremes of the PRS was both reported previously [12, 14, 72–75]. In conclusion, although there is not direct benefit of including genetic risk factors into diagnostic work-up at this time, their importance is clearly growing. This recommendation may quickly change if disease-modifying treatments (DMT) become available. These DMTs likely need to be given early in the clinical trajectory and genetic tests can be used to screening for individuals at high risk of cognitive decline. This is in contrast with neuroimaging [76, 77] and CSF/blood-based biomarkers [78–80] which could aid decision making in more intermediate stages and cases with multiple pathologies. Testing for specific risk factors, such as *APOE* [81, 82] or *TREM2* [83, 84] may also be initiated if gene-specific treatments can be offered, first in clinical trials and later hopefully as precision medication. In fact, *APOE* testing will likely become standard to predict the risk of amyloid related imaging abnormalities (ARIA), a side effect of DMT.

## Conclusions

Overall, DNA diagnostics represent a promising approach to enhancing the precision of clinical dementia diagnosis, especially in memory clinic settings. Incorporating testing for causal factors in memory clinics could benefit patients immediately by providing personalized treatment following an accurate genetic diagnosis. We anticipate that the implementation of risk variant screening will become integral to clinical care as disease-modifying treatments become available.

## Supplementary Information

Below is the link to the electronic supplementary material.

Supplementary Material 1

Supplementary Material 2

## Data Availability

Data can be accessed upon request.
