# Supplementary material for: Genetic testing of common and rare variants in dementia patients from a memory clinic: Dementia-related genetic testing in memory clinic
Source: Alzheimers Res Ther. 2025 Oct 14;17:225. doi: 10.1186/s13195-025-01854-z (PMC12522692; doi:10.1186/s13195-025-01854-z)
Supplement: Supplementary file 1 — Supplementary Material 1 [file 13195_2025_1854_MOESM1_ESM.docx]

**Supplementary Information**

**Genetic testing of common and rare variants in dementia patients from a memory clinic**

**Dementia-Related Genetic Testing in Memory Clinic**

**Supplementary methods**

*Genotyping and Imputation Procedures*

Individuals were genotyped using Illumina Global Screening Array (GSAsharedCUSTOM_20018389_A2) as part of the European Alzheimer’s and Dementia Biobank (EADB). Genetic variants and imputation were determined by standard quality control methods. For more details and methods used for genotyping and quality controls applied see previous publications^1^. In sum, individuals without high-quality genotyping (call rate >98%) and sex mismatches were removed. Variants with call rate below 98% or deviation from the Hardy–Weinberg equilibrium (HWE) p<1×10^−06^ were also excluded from the analysis. All autosomal variants were submitted to the Trans-Omics for Precision Medicine (TOPMed) imputation server^2–4^ (build GRCh38). Relatives were not excluded from the study in order to reflect the real-world clinical setting. We observed 5 sibling pairs and 4 distant relatives IBS<0.2 (identical by state) and non-European descent represent the 9% of the total cohort.

*Whole exome sequencing*

We collected whole exome sequencing data using the Agilent v4 or v6 kits (~58Mb target region). Sequencing was done on Illumina NovaSeq 6000 (2x150 basepair reads) and samples had at least 8Gb raw data per sample. A uniform pipeline was used to process all samples as previously described^5^. Raw sequencing was processed relative to the GRCh37 reference genome, the read alignments of possible chimeric origin were filtered and a GATK-based pipeline was used to call variants, while correcting for estimated sample contamination percentages. Samples were included in the datasets after they passed a stringent QC pipeline: samples were removed when they had high missingness, high contamination, a discordant genetic sex annotation, high numbers of new variants (with reference to dbSNP v.150), deviating heterozygous/homozygous or transition/transversion ratios. We did not exclude individuals of non-European ancestry nor family members, as they are part of routine clinical care and including them reflects real-world diagnostic settings.

Subsequently, we selected variants in autosomal protein-coding genes that were part of the Ensembl basic set of protein coding transcripts (Gencode v19/v2926, see publication^5^) and that were annotated by the Variant Effect Predictor (VEP)^6^ (version 94.542). Only protein coding missense and loss-of-function (LOF) variants were considered (LOF: nonsense, splice acceptor/donor or frameshifts). Missense and LOF variants were required to have respectively a ‘moderate’ and ‘high’ VEP impact classification. Then, missense variants were prioritized using Rare Exome Variant Ensemble Learner (REVEL)^7^ annotation obtained from DBNSFP4.1a^8^ and LOF variants were prioritized using LOFTEE^9^ (version 1.0.2).

*Measuring the APP duplications and C9orf72 hexanucleotide repeat lengths*

The detection of *APP* duplications was an array-based test in research setting and a Multiplex ligation-dependent probe amplification (MLPA)-based in clinical procedures.

We used the standard methods described in the Renton et al.^10^, to measure the *C9orf72* repeat lengths. Repeat expansions of with minor modifications. All expansions, and if only 1 allele was present, were followed-up by either repeat-primed PCR or a commercial kit^11^ (AmplideX PCR/CE C9orf72 Kit, Asuragen). Hexanucleotide repeat lengths over 30 were considered pathogenic. In the implementation cohort *C9orf72* repeat lengths were determined with a commercial kit (AmplideX PCR/CE C9orf72 Kit, Asuragen). For more information, see our previous publication^12^.

**Supplementary results**

*AD-PRS*

After the stratification of the common AD-PRS into quintiles based on the SCD-PRS distribution (category 3), we found and enrichment within AD patients into the highest risk quintile (39%) compare to the lowest PRS quintile (4%). However, although we did not find significant difference in terms of age at entry, sex, positive family history, *APOEɛ4* or rare variant carriers between extreme quintiles of common AD-PRS risk groups (without *APOE*) (Supplementary Fig. 7), we did see a greater number of *APOEɛ4* and rare variant carriers in the highest PRS group (Supplementary Fig. 8).

When we looked at the AD-PRS we found significantly higher differences between AD and SCD (nAD=354, nSCD=216, p=4.25E-07, Supplementary Fig. 6A). This association was independent of *APOEɛ4* as there it is not included in the PRS. Thus, regression models adjusted by age, sex, *APOEɛ4* and PRS-TREM2 interaction confirm the association between the PRS and AD (OR=1.62[1.32-2.00], p=5.90E-06, Supplementary Table 6).

The multinomial regression model showed a significant risk effect of the PRS adjusted by age in AD (OR=2.0[1.64-2.44], p=8.9E-12), LBD (OR=1.8[1.23-2.64], p=2.4E-03), and non-significant (p>0.05) effect for the other phenotypes (FTD, other dementia, psychiatric diseases and MCI) with SCD as a reference (Supplementary Fig. 9). These results indicate that the AD-PRS allows for the differentiation of the AD clinical phenotype with a higher risk compared to the SCD population.

How it’s already known, the *APOE* influences the AD risk in a dose-dependent manner. In our cohort, compared to the reference ε3ε3 genotype, one ɛ4 allele increases AD risk by 2-fold (OR=2.45[1.60-3.75], p=3.6E-05) while AD risk is increased to 4-fold in individuals with two ɛ4 alleles (OR=3.93[2.02-7.67], p=6.0E-05). In the same way, when we stratify the AD-PRS in quintiles we found a significant risk increased in the high-risk quintile (q5) compared to the reference (q3), reaching almost 2 times more risk of AD (OR=1.81[1.03-3.19], p=4.1E-02, Supplementary Fig. 6C).

Finally, when examining the age at entry distributions, as expected, the group of individuals with dementia who comes earlier to the clinic exhibited a higher prevalence of *APOEε4* carriers, rare variants linked to AD, a family history of dementia, and a higher risk PRS compared to their older counterparts and individuals with normal cognitive function (Supplementary Fig. 10).

**References**

1. Bellenguez, C. *et al.* New insights into the genetic etiology of Alzheimer’s disease and related dementias. *Nature Genetics 2022* 1–25 (2022) doi:10.1038/s41588-022-01024-z.

2. Taliun, D. *et al.* Sequencing of 53,831 diverse genomes from the NHLBI TOPMed program. *bioRxiv* vol. 2 563866 Preprint at https://doi.org/10.1101/563866 (2019).

3. Das, S. *et al.* Next-generation genotype imputation service and methods. *Nat Genet* **48**, 1284–1287 (2016).

4. Fuchsberger, C., Abecasis, G. R. & Hinds, D. A. minimac2: faster genotype imputation. *Bioinformatics* **31**, 782–784 (2015).

5. Holstege, H. *et al.* Exome sequencing identifies rare damaging variants in ATP8B4 and ABCA1 as risk factors for Alzheimer’s disease. *Nature Genetics 2022* 1–9 (2022) doi:10.1038/s41588-022-01208-7.

6. Liu, X., Li, C., Mou, C., Dong, Y. & Tu, Y. dbNSFP v4: a comprehensive database of transcript-specific functional predictions and annotations for human nonsynonymous and splice-site SNVs. *Genome Med* **12**, 1–8 (2020).

7. Karczewski, K. J. *et al.* The mutational constraint spectrum quantified from variation in 141,456 humans. *Nature 2020 581:7809* **581**, 434–443 (2020).

8. Benjamini, Y. & Hochberg, Y. Controlling the False Discovery Rate: A Practical and Powerful Approach to Multiple Testing. *Journal of the Royal Statistical Society: Series B (Methodological)* **57**, 289–300 (1995).

9. Holm, S. A Simple Sequentially Rejective Multiple Test Procedure. *Scandinavian Journal of Statistics* (1979) doi:10.2307/4615733.

10. Renton, A. E. *et al.* A hexanucleotide repeat expansion in C9ORF72 is the cause of chromosome 9p21-linked ALS-FTD. *Neuron* **72**, 257–268 (2011).

11. Reus, L. M. *et al.* Genome-wide association study of frontotemporal dementia identifies a C9ORF72 haplotype with a median of 12-G4C2 repeats that predisposes to pathological repeat expansions. *Translational Psychiatry 2021 11:1* **11**, 1–8 (2021).

12. Van Der Lee, S. J. *et al.* Prevalence of Pathogenic Variants and Eligibility Criteria for Genetic Testing in Patients Who Visit a Memory Clinic. *Neurology* **104**, (2025).

**Supplementary Figures**

**
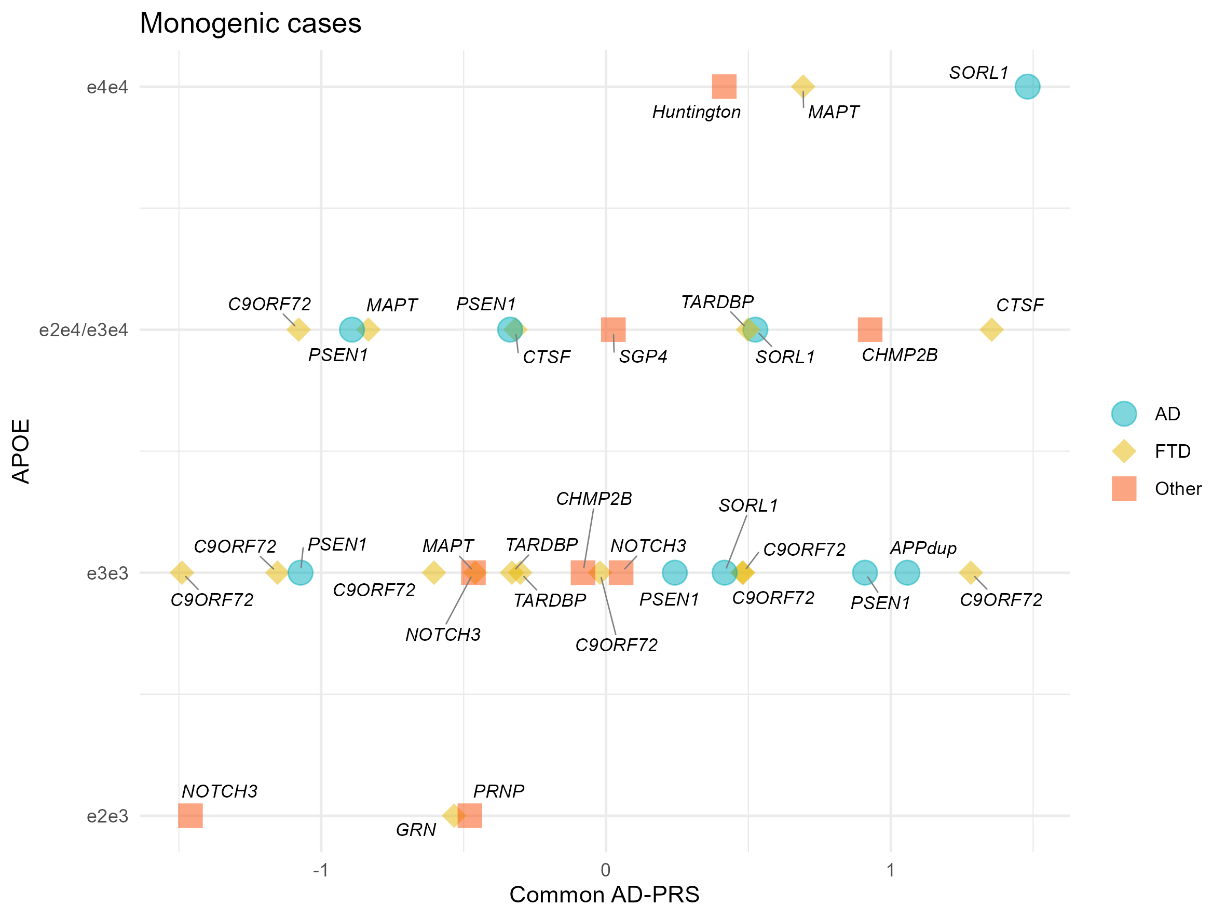
**

**Supplementary Figure 1:** Common AD-PRS distribution by *APOE* genotype from the monogenic carriers’ phenotypes.


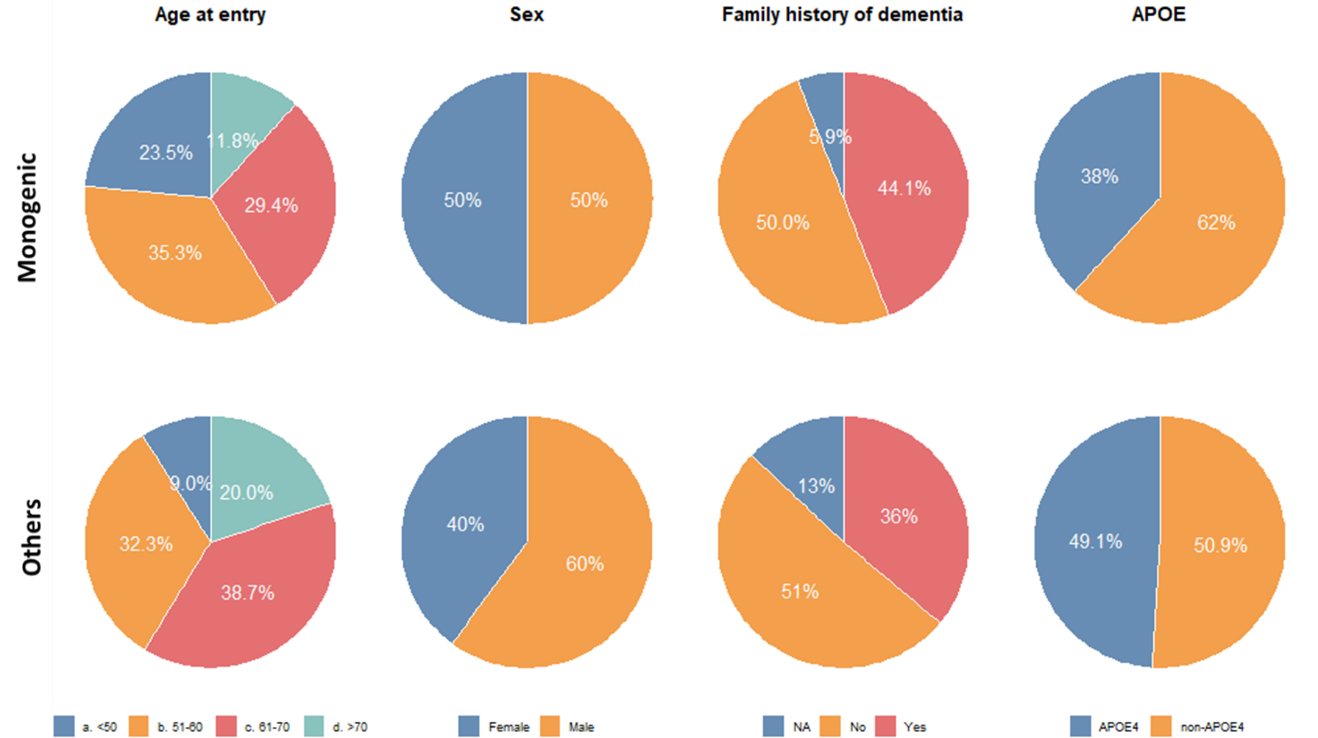


**Supplementary Figure 2:** Demographic comparisons between monogenic carriers and the rest of individuals from our clinical cohort (n=998).

**
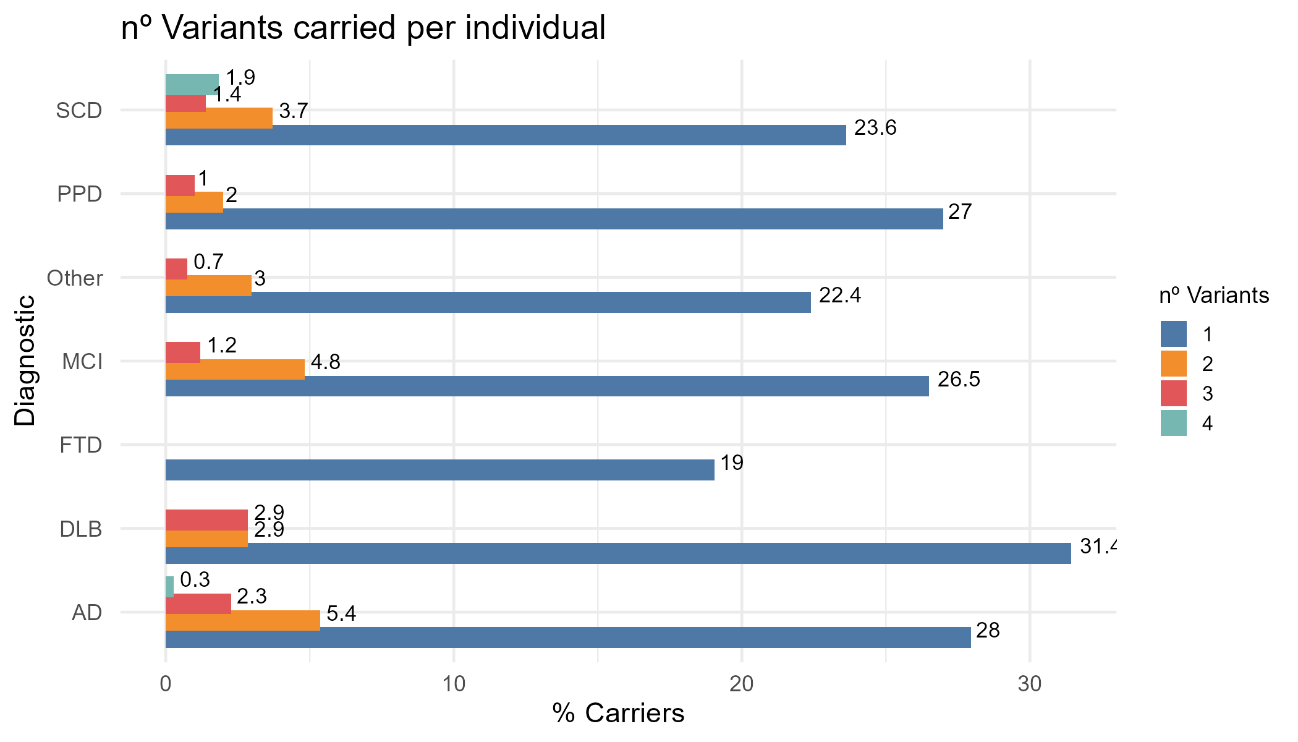
Supplementary Figure 3:** Distribution of rare variants per individual from our clinical cohort (n=964).


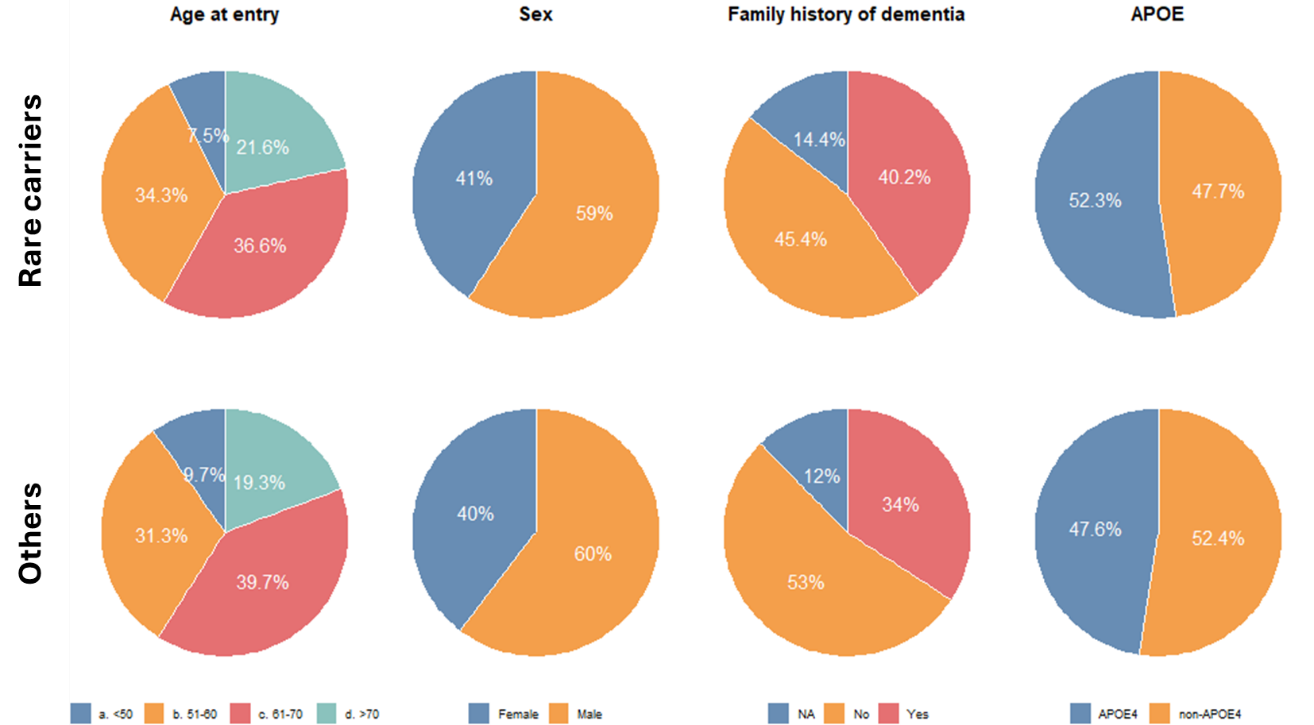


**Supplementary Figure 4:** Demographic comparisons between the carriers of rare moderately penetrant risk/modifier genes for AD and the rest of individuals from our clinical cohort (n=964).


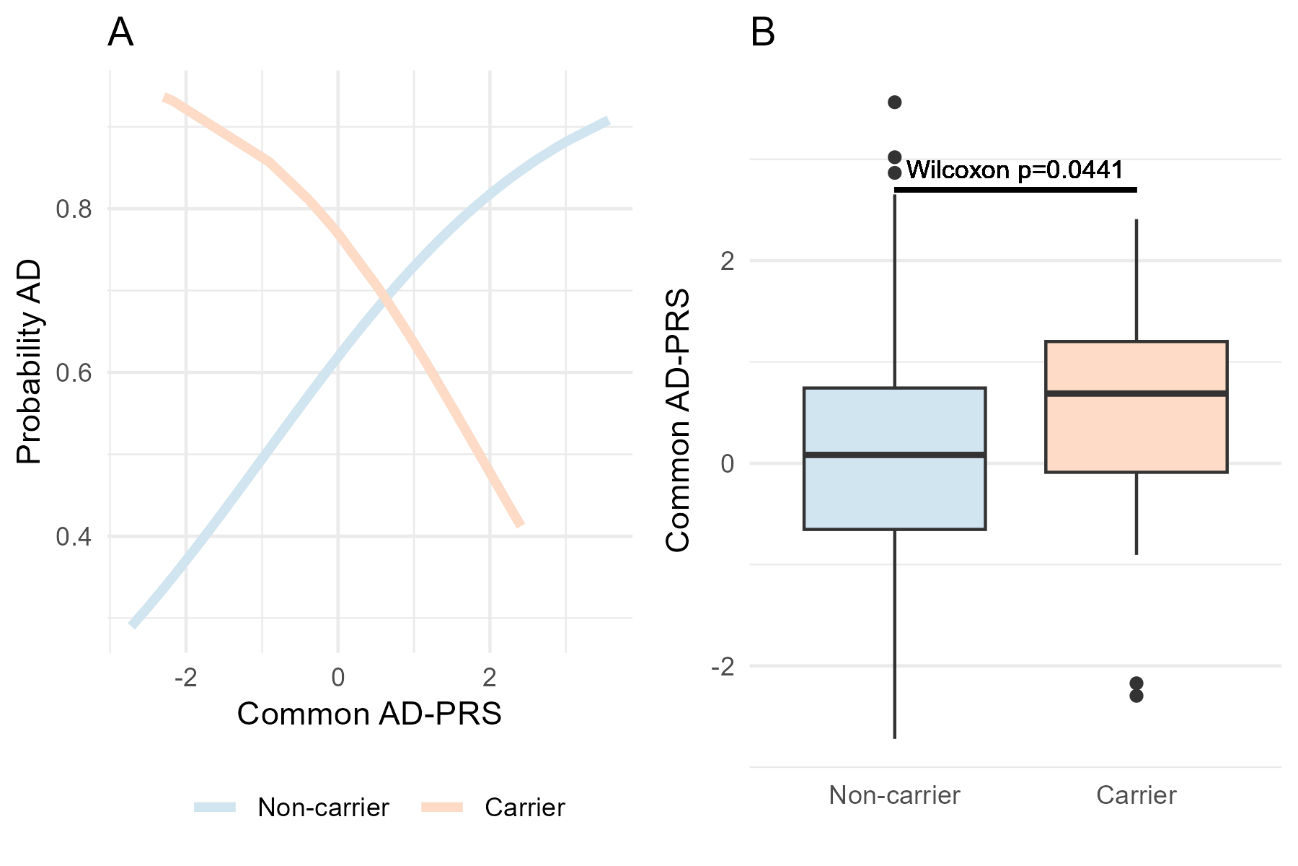


**Supplementary Figure 5:** Stratification of *TREM2* rare variant carriers. A) Interaction between common AD-PRS and *TREM2* rare variant carrier status, B) Distribution of AD-PRS values in *TREM2* rare variant carriers versus non-carriers (boxplot).


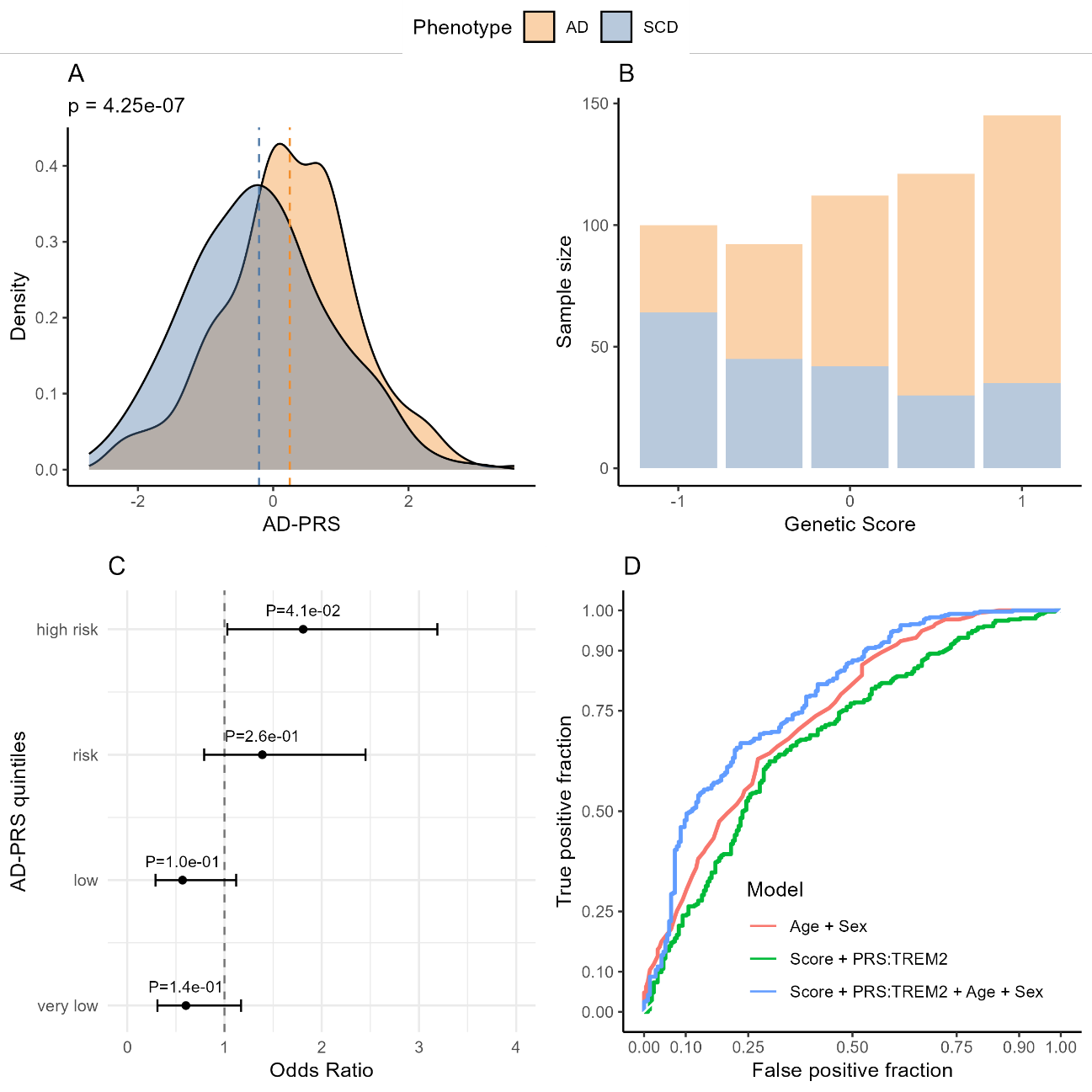


**Supplementary Figure 6:** Genetic AD risk distribution and stratification. A) Common AD-PRS (without *APOE*), B) Genetic Score stratification, C) The AD risk stratification by quintiles of the common AD-PRS (without *APOE*) compared to the reference quintile (q3) adjusted by age and sex, D) Area under the receiver operating characteristic curve (AUC) in SCD vs AD (n=570). ‘PRS:TREM2’ represents the interaction between common AD-PRS and carrying a rare *TREM2* variant.


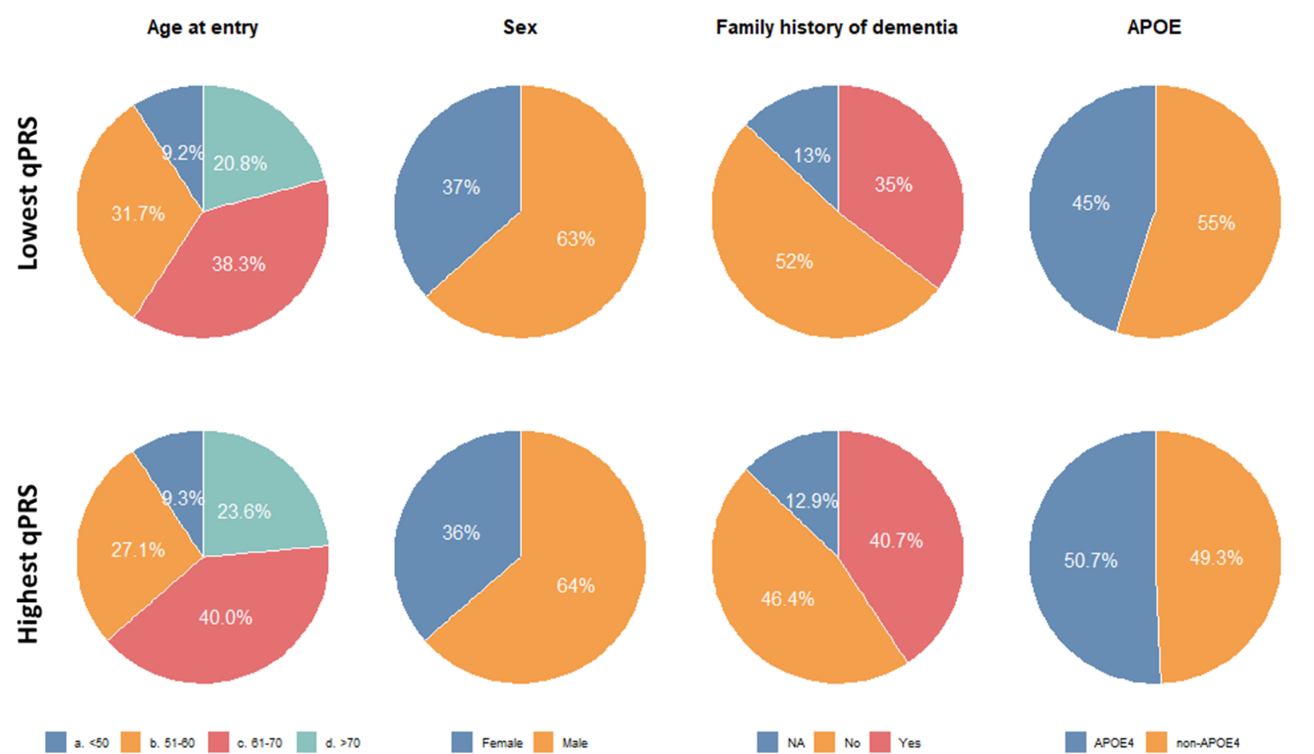


**Supplementary Figure 7:** Demographic comparisons between the lowest and highest AD-PRS quintiles (without *APOE*) individuals in our clinical cohort (n=964).


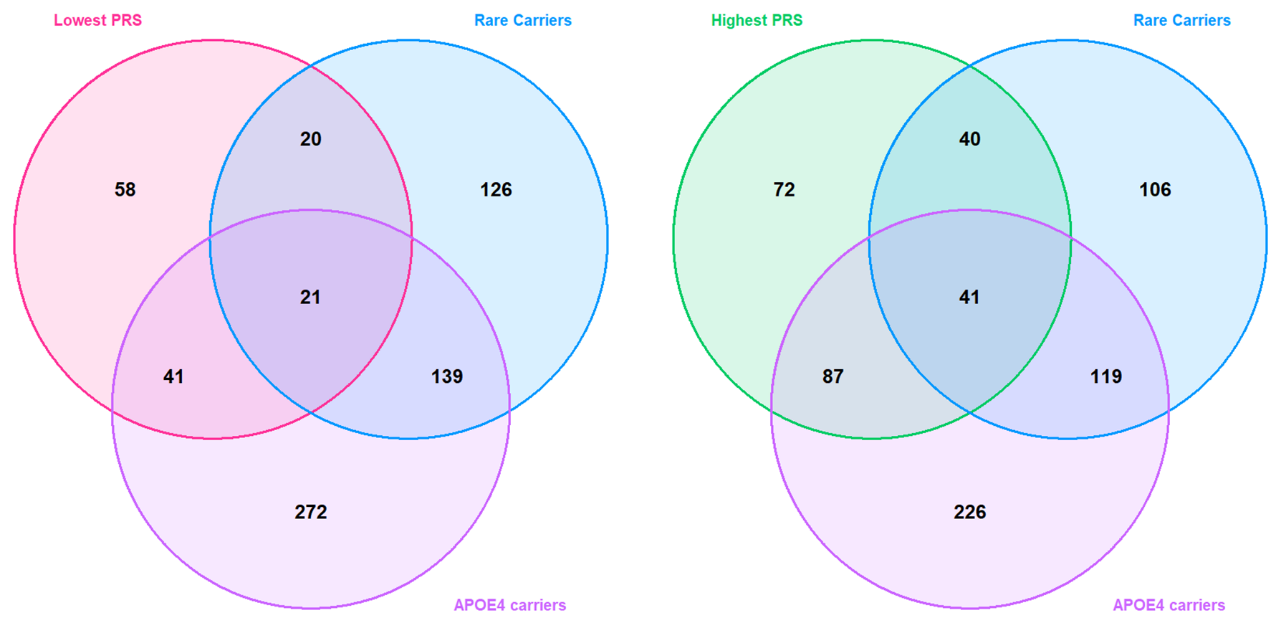


**Supplementary Figure 8:** Venn diagram between the three different AD genetic risk groups: common polygenic risk score, *APOEε4* carriers and carriers of rare moderately penetrant risk/modifier genes for AD.

**
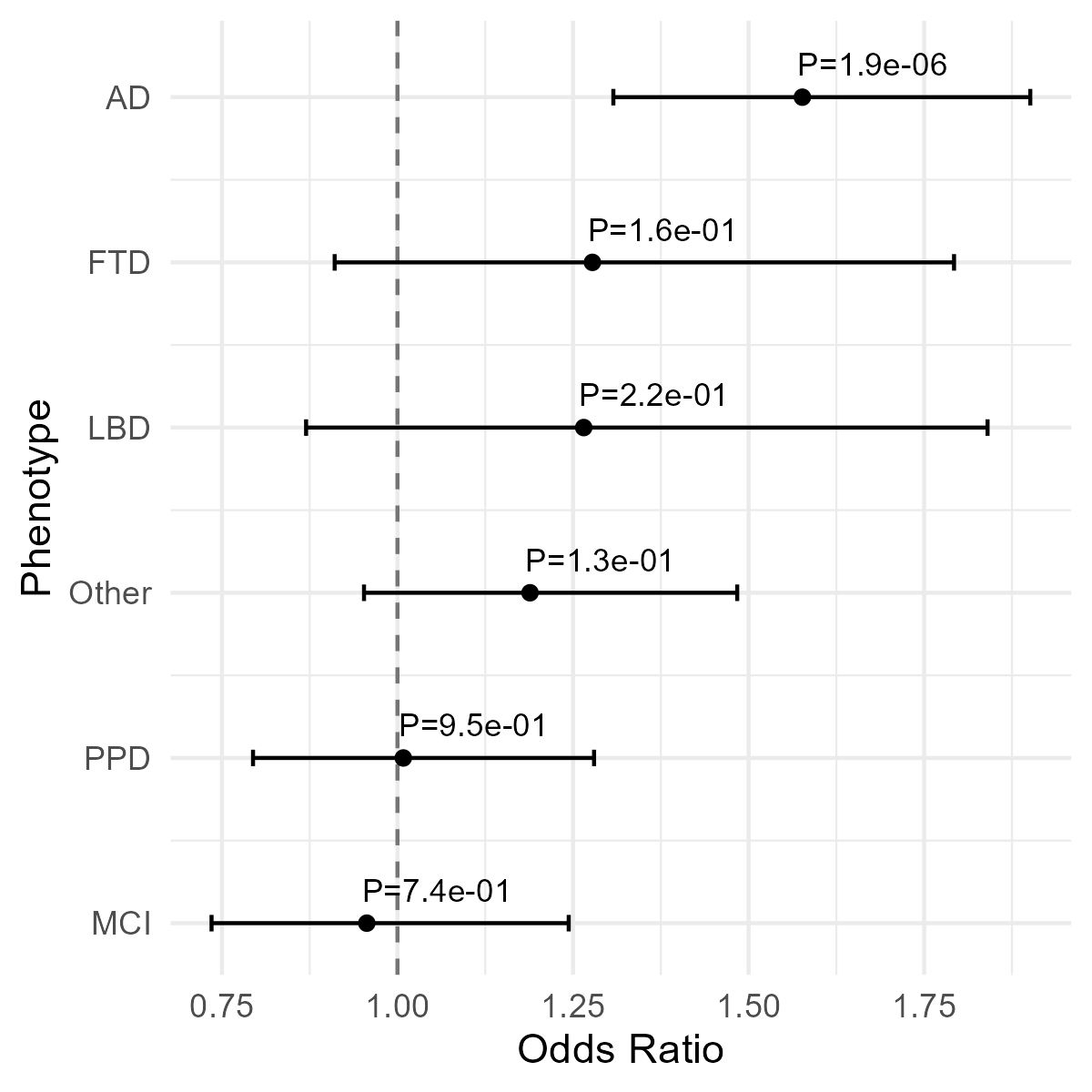
**

**Supplementary Figure 9:** Common AD-PRS and phenotypes associations. Multinomial regression model with subjective cognitive decline (SCD) as a reference category and adjusted by age. AD-PRS without *APOE*.

**
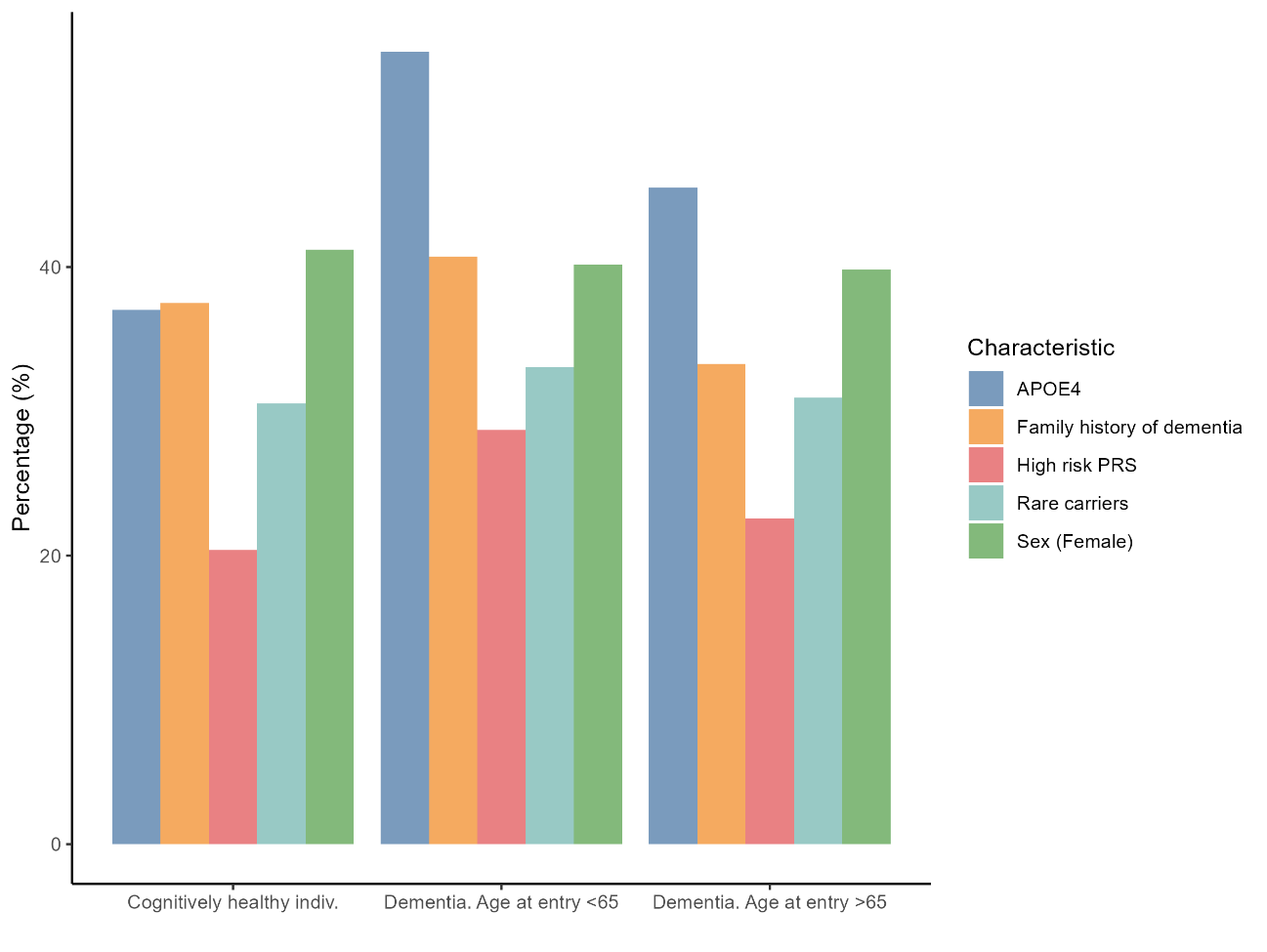
**

**Supplementary Figure 10:** Percentage of cognitively healthy and dementia individuals with different characteristics based on their age at entry. High risk PRS was calculated based on the quintile PRS distribution (q5).

**Supplementary Tables**

| **Gene name** | **Gene name** | **Gene name** |
| --- | --- | --- |
| Hexanucleotide *C9ORF72* repeat | *ALS2* (NM_020919.3) | *ANG* (NM_001145.4) |
| *APOE* (NM_001302688.1) | *APP* (NM_000484.3) | duplications of *APP* (NM_000484.3) |
| *ATP7B* (NM_000053.3) | *C19ORF12* (NM_001031726.3) | *C9ORF72* (NM_001256054.2) |
| *CHCHD10* (NM_001301339.1) | *CHMP2B* (NM_014043.3) | *CLN3* (NM_001042432.1) |
| *CLN5* (NM_006493.2) | *CP* (NM_000096.3) | *CSF1R* (NM_005211.3) |
| *CTSD* (NM_001909.4) | *CTSF* (NM_003793.3) | *EIF4G1* (NM_182917.4) |
| *ERBB4* (NM_005235.2) | *FUS* (NM_004960.3) | *GRN* (NM_002087.3) |
| *HNRNPA1* (NM_031157.3) | *HNRNPA2B1* (NM_031243.2) | *HTRA1* (NM_002775.4) |
| *ITM2B* (NM_021999.4) | *MAPT* (NM_005910.5) | *NOTCH3* (NM_000435.2) |
| *NPC1* (NM_000271.4) | *NPC2* (NM_006432.3) | *OPTN* (NM_001008211.1) |
| *PDGFB* (NM_002608.3) | *PDGFRB* (NM_002609.3) | *PPT1* (NM_000310.3) |
| *PRKAR1B* (NM_001164761.1) | *PRNP* (NM_000311.3) | *PSEN1* (NM_000021.3) |
| *PSEN2* (NM_000447.2) | *PSENEN* (NM_172341.3) | *SERPINI1* (NM_005025.4) |
| *SETX* (NM_015046.5) | *SIGMAR1* (NM_005866.3) | *SLC20A2* (NM_001257180.1) |
| *SNCA* (NM_000345.3) | *SNCB* (NM_001001502.2) | *SOD1* (NM_000454.4) |
| *SORL1* (NM_003105.5) | *SPG11* (NM_025137.3) | *SQSTM1* (NM_003900.4) |
| *TARDBP* (NM_007375.3) | *TBK1* (NM_013254.3) | *TREM2* (NM_018965.3) |
| *TYROBP* (NM_003332.3) | *UBQLN2* (NM_013444.3) | *VCP* (NM_007126.3) |
| *VPS13A* (NM_033305.2) | *XPR1* (NM_004736.3) |  |

**Supplementary table 1:** Genes in the dementia gene panel for the identification of monogenic cause for dementia.

| *Logistic Regression* | |  |  |  |  |
| --- | --- | --- | --- | --- | --- |
|  | ***Estimate*** | ***Std.Error*** | ***z value*** | ***Pr(>\|z\|)*** |  |
| (Intercept) | -6.631 | 0.839 | -7.905 | 2.68E-15 | *** |
| PRS-AD | 0.432 | 0.104 | 4.144 | 3.41E-05 | *** |
| PRS-rare | 0.100 | 0.092 | 1.082 | 0.279 |  |
| Sex (Male) | -0.384 | 0.203 | -1.894 | 0.058 | . |
| Age | 0.110 | 0.013 | 8.221 | <2.00E-16 | *** |
| APOE4 carrier | 0.960 | 0.201 | 4.782 | 1.73E-06 | *** |
| PRS_AD:PRS_rare | -0.162 | 0.086 | -1.879 | 0.060 | . |

**Supplementary Table 5:** Logistic regression in SCD (n=216) vs AD (n=354) individuals with interaction between common AD-PRS and rare-PRS.

| Model | OR [95%CI] | P-value |
| --- | --- | --- |
| AD-PRS | 1.56[1.31-1.86] | 6.16E-07 |
| AD-PRS + PRS:TREM2 | 1.66[1.38-2.00] | 6.47E-08 |
| AD-PRS + PRS:TREM2 + *APOE4* | 1.62[1.34-1.96] | 6.66E-07 |
| AD-PRS + PRS:TREM2 + *APOE4* + Age + Sex | 1.62[1.32-2.00] | 5.90E-06 |
| Genetic Score | 1.80 [1.50-2.17] | 6.61E-10 |
| Genetic Score + PRS:TREM2 | 1.90[1.57-2.31] | 7.09E-11 |
| Genetic Score + PRS:TREM2 + Age + Sex | 1.82[1.48-2.23] | 7.78E-09 |
| Genetic Score risk + PRS:TREM2 + Age + Sex | 1.97[1.60-2.44] | 3.15E-10 |

**Supplementary Table 6:** Logistic regression association models in SCD (n=216) vs AD (n=354) individuals. ‘PRS:TREM2’ represents the interaction between common AD-PRS and carrying a rare *TREM2* variant. ‘Genetic Score risk’ is the genetic score combining common variant PRS, *APOE* and rare variant burden from five risk genes (*SORL1, TREM2, ABCA7, ABCA1, ATP8B4*).

| Score | AD | LBD | FTD | MCI | Other | PPD | SCD |
| --- | --- | --- | --- | --- | --- | --- | --- |
| q1 (-1) | 14 | 1 | 5 | 12 | 15 | 13 | 43 |
| q2 (-0.5) | 43 | 5 | 12 | 20 | 34 | 22 | 44 |
| q3 (0) | 54 | 7 | 9 | 16 | 32 | 28 | 43 |
| q4 (0.5) | 105 | 10 | 10 | 17 | 24 | 21 | 42 |
| q5 (1) | 138 | 12 | 6 | 18 | 29 | 16 | 44 |

**Supplementary Table 7:** Genetic score quintile distributions per phenotype, adjusted to SCD population.
